# Inflammatory complications of CGRP monoclonal antibodies: a case series

**DOI:** 10.1186/s10194-021-01330-7

**Published:** 2021-10-09

**Authors:** Jason C. Ray, Penelope Allen, Ann Bacsi, Julian J. Bosco, Luke Chen, Michael Eller, Hock Kua, Lyndell L. Lim, Manjit S. Matharu, Mastura Monif, Martin Ruttledge, Richard J. Stark, Elspeth J. Hutton

**Affiliations:** 1 Department of Neurology, Alfred Hospital, Commercial Melbourne 3004, Melbourne, Australia; 2 Department of Neurology, Austin Health, 145 Studley Road, 3084 Heidelberg, Germany; 3 Department of Neuroscience, Monash University, Vic, Melbourne, 3004 Australia; 4 Centre for Eye Research Australia, Royal Victorian Eye and Ear Hospital, East Melbourne, Australia; 5 Department of Surgery (Ophthalmology), University of Melbourne, Parkville, Australia; 6 Integrated Specialist Medical Care, Sydney, Australia; 7 Department of Allergy, asthma and clinical immunology, Alfred Hospital, Commercial Road 3004, Melbourne, Australia; 8 Central Clinical School, Faculty of Medicine Nursing and Health Sciences, Monash University, Melbourne, Australia; 9 Otoneurology Diagnostic Unit, Alfred Hospital, Commercial Rd 3004, Melbourne, VIC Australia; 10 Department of Neurology, Monash Medical Centre, Vic, Melbourne, Australia; 11 School of Clinical Sciences, Monash University, Vic, Melbourne, Australia; 12 Department of Pathology, Monash Medical Centre, Vic, Melbourne, Australia; 13 University College London (UCL) Queen Square Institute of Neurology and The National Hospital for Neurology and Neurosurgery, Queen Square, University College London, Gower Street WC1E 6BT, London, UK; 14 Department of Neurology, Royal Melbourne Hospital, Vic, Parkville, 3050 Australia; 15 MS and Neuroimmunology Department, Alfred Hospital, Vic, Melbourne, 3004 Australia; 16 Consultant Neurologist & Headache Clinical Lead, Beaumont Hospital, Beaumont Road, Dublin, Ireland

**Keywords:** Migraine, CGRP, CGRP receptor antagonists, Monoclonal antibodies, Autoimmune diseases, Drug-related side effects and adverse reactions

## Abstract

**Background:**

Calcitonin gene-related peptide (CGRP) is expressed throughout the body and is a known mediator of migraine, exerting this biological effect through activation of trigeminovascular, meningeal and associated neuronal pathways located in close proximity to the central nervous system. Monoclonal antibodies (mAb) targeting the CGRP pathway are an effective new preventive treatment for migraine, with a generally favourable adverse event profile. Pre-clinical evidence supports an anti-inflammatory/immunoregulatory role for CGRP in other organ systems, and therefore inhibition of the normal action of this peptide may promote a pro-inflammatory response.

**Cases:**

We present a case series of eight patients with new or significantly worsened inflammatory pathology in close temporal association with the commencement of CGRP mAb therapy.

**Conclusion:**

This case series provides novel insights on the potential molecular mechanisms and side-effects of CGRP antagonism in migraine and supports clinical vigilance in patient care going forward.

## Background

Calcitonin gene-related peptide (CGRP) is an important neuropeptide in migraine pathophysiology. During a migraine attack, activation of first-order trigeminovascular neurons by various mechanisms results in sensitisation and activation of second and third order neurons, and the perception of migraine pain [1]. Activation of the trigeminovascular system results in the release of, amongst other vasoactive peptides, CGRP, which in turn contributes to the vasodilation of intracranial vessels, activation of local neuro-inflammatory cascades and the genesis of a migraine attack [1]. CGRP is released in response to transient receptor potential vanilloid 1 (TRPV1) receptor activation, as well as tryptase, bradykinin and prostaglandin release [2–4]. CGRP inhibition by specific monoclonal antibodies (mAb) effectively controls migraines in a significant proportion of patients, as demonstrated in the phase II and III trials [5, 6].

Favourable adverse event profiles have been reported in these trials, as well as open label extension and ‘real-world’ studies [5–8]. This preliminary safety data is promising, but there needs to be ongoing vigilance for new safety events. For example, approximately 30% of all new medications licensed by the FDA between 2001 and 2010 went on to have additional post-marketing safety events [6, 8].

CGRP and CGRP receptors are expressed widely throughout the body, and as such there are several theoretical off-target effects of long-term inhibition, and these have been summarised in several recent review articles [6, 9]. The most significant possible consequences include inhibition of angiogenesis, inhibition of vasodilation, the possibility of osteoporosis, disruption of gastrointestinal mucosal integrity and constipation [6, 10, 11]. To date, there have been case reports of probable migraine-related stroke, polyarthralgia, and reversible cerebral vasoconstriction syndrome possibly related to CGRP mAb use [12]. In addition, the various effects of CGRP on the immune system, and the potential impact of CGRP inhibition has been thoroughly summarised by Assas [10, 13]. Here, we present for the first time, a case series of systemic inflammatory disorders following CGRP inhibition.

## Case series

**Fig. 1:**
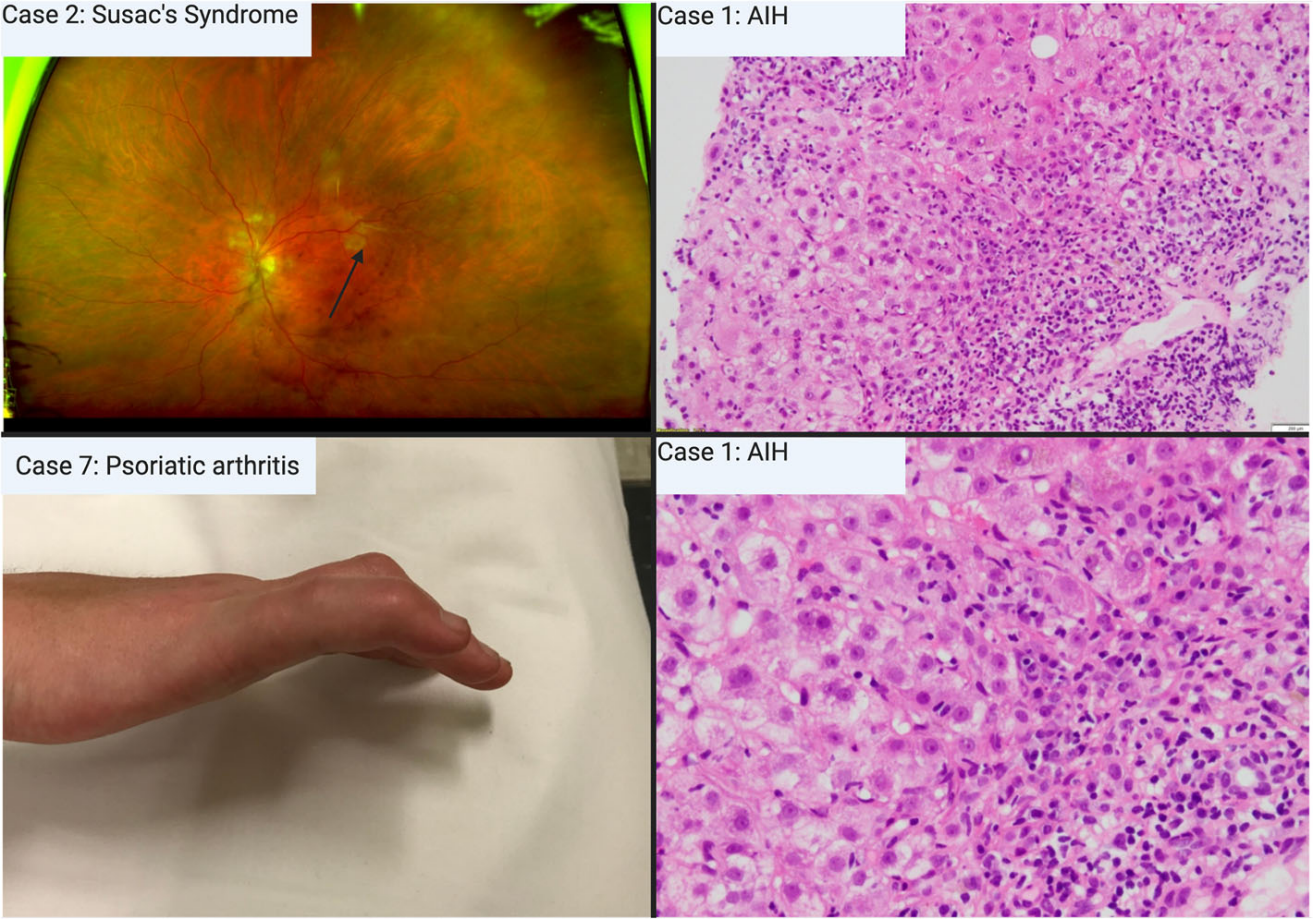
*Case images of inflammatory complications of CGRP inhibition.* Upper left: fluorescein angiography of case 2 (Susac’s syndrome) showing branch retinal artery occlusion. Lower left: active synovitis of psoriatic arthritis in case 7 Upper right: 10x magnification of liver biopsy showing changes of interface hepatitis in case 1, lower right: 20x magnification of liver biopsy further highlighting plasma cells at interface of hepatocytes in case 1 autoimmune hepatitis (AIH)

A total of eight cases (7 women, 1 man) were identified by clinicians practising in Australia and Ireland in tertiary and private headache clinics during 2019 and 2020. The mean age of onset of inflammatory symptoms was 43 (SD 14.17, range 20–67) (Table 1). Three of the eight cases had a history of previously well controlled or quiescent rheumatological or dermatological disease prior to commencement of therapy. Reflecting local availability, six patients were treated with erenumab, (one switching later to fremanezumab) and two received galcanezumab. Six cases developed a de-novo inflammatory condition post exposure (median 88 days, IQR 71–105) to a CGRP mAb, while the remaining two patients had a significant and unexpected exacerbation of their previously very well controlled immune-mediated disease (Fig. 1). The clinical manifestations were significant enough to warrant medical intervention and systemic immunosuppression in six of the cases. There were no other relevant potential triggers identified such as infection, medication or neoplasia during the follow-up. Only one patient received a further dose of a CGRP mAb after their complication became apparent (case 2).

**Table 1:** Patients with temporally-associated inflammatory complications of CGRP inhibition

Patient	Comorbidities	Concomitant medications	Headache history	CGRP mAb treatment	Response to CGRP mAb	Complication (Onset of Cx)	Diagnosis of complication	Management of complication
Case 1 56 M	BMI 28.4 kg/m^2^ Rheumatoid arthritis (quiescent), anxiety, dyslipidaemia, pulmonary fibrosis	Rosuvastatin, atenolol, fenofibrate	CM 3 oral preventatives, onaB	Erenumab, single dose	–	Autoimmune hepatitis (D14 of Rx)	Specialist diagnosis, biopsy proven	CGRP agent ceased. Stabilised with steroid and azathioprine
Case 2 67F	BMI 23.5 kg/m^2^ Chronic fatigue syndrome, fibromyalgia, hypertension, constipation	Candesartan, pregabalin, esomeprazole, clomipramine, naratriptan	CM 11 oral preventatives, onaB	Erenumab, 15 months	> 75% reduction MMD	Ocular Susac’s syndrome (12 months of Rx)	Specialist diagnosis	CGRP agent ceased. No further flares, or progression to brain/auditory involvement with IVIg, aspirin, prednisolone, mycophenolate.
Case 3 44F	BMI 20.8 kg/m^2^	Zolmitriptan	CM 7 oral preventatives, onaB	Erenumab, single dose	> 75% reduction MMD	DRESS Syndrome (D26 of Rx)	Specialist diagnosis, biopsy proven	CGRP agent ceased. Resolved within four weeks tapering 1 mg/kg prednisolone.
Case 4 32F	BMI 35 kg/m^2^	Topiramate	CM 3 oral preventatives	Erenumab, 6 months transitioned to Fremanezumab, 5 months	> 75% reduction MMD	Granulomatosis with polyangiitis (5 months of Rx)	Specialist diagnosis, biopsy proven	CGRP agent ceased. Complication ongoing with prednisolone and methotrexate.
Case 5 20F	BMI 18.9 kg/m^2^ IgG4 disease, GORD, osteoporosis, adrenal insufficiency	10 mg Prednisolone, gabapentin, calcitriol, propranolol, tapentadol, nortriptyline, CBD oil, omeprazole	CM 6 oral preventatives, onaB	Galcanezumab, single dose	–	Severe polyarthralgia (D4 of Rx)	Specialist diagnosis	CGRP agent ceased. Complication improved 30% but ongoing
Case 6 45F	BMI 32.3 kg/m^2^ Psoriasis, chronic fatigue syndrome, Lyme disease	Roxithromycin, tinidazole, minocycline, n-acetyl cysteine, naratriptan, botulinum toxin, rosuvastatin	CM 3 oral preventatives, onaB	Galcanezumab, single dose	None	Severe exacerbation of psoriasis (PASI score 16) (D2 of Rx)	Specialist diagnosis, biopsy proven	CGRP agent ceased. Complication improved (PASI 2) with steroid cream and methotrexate.
Case 7 41F	BMI 25.6 kg/m^2^ Psoriatic arthritis (previously controlled > 5 years on adalimumab), osteopenia	Adalimumab, pantoprazole, gabapentin, verapamil, amitriptyline, fenofibrate, naratriptan	CM 5 oral preventatives, onaB	Erenumab, single dose	> 50% reduction MMD	Flare of psoriatic arthritis (5 months of Rx)	Specialist diagnosis	CGRP agent ceased. Complication improving with steroids and change in biological therapy.
Case 8 46F EH	BMI 31 kg/m^2^ Asthma	Propranolol, microgynon, Symbicort, PRN Maxalt	CM 7 oral preventatives, onaB	Erenumab, 18 months	> 50% reduction MMD	Urticarial eczema (16 months of Rx)	Specialist diagnosis	CGRP agent ceased. Complication improving with topical steroids, emollients, UVB therapy

CBD: cannabidiol, CGRP: Calcitonin Gene Related Peptide, DRESS: Drug reaction with eosinophilia and systemic symptoms, GORD: Gastro-oesophageal reflux disease, IVIg: intravenous immunoglobulin, onaB: onabotulinumtoxinA, PASI: Psoriasis area and severity index, PRN: pro re nata (when necessary), Rx: Treatment, UVB: ultraviolet-B, CM; chronic migraine, BMI; Body mass index

## Discussion

CGRP pathway inhibition is clearly a significant therapeutic development for migraine patients, with demonstrated efficacy in both clinical trials and real-world studies. CGRP has additional effects beyond migraine neurobiology however, and the effects that it has on the immune system are diverse. Centrally, CGRP induces migraine indirectly via peripheral afferents, and has a role in the promotion of pro-inflammatory cytokines [14]. In experimental autoimmune encephalomyelitis (EAE), the experimental model of multiple sclerosis, CGRP has also been shown to inhibit microglia activation, and CGRP-expressing dendritic cells have been shown to suppress the development of the condition [15, 16]. However, there is no evidence that that CGRP monoclonal antibodies cross the blood-brain barrier in significant amounts and therefore their use is unlikely to have a direct effect on microglia or other central neural pathways.

Peripherally, CGRP expressing nerve fibres have been identified in lymphoid organs, bone marrow, skin, lungs and intestine, and serum CGRP levels are increased as a regulatory response in systemic inflammation, such as post-operative sepsis [2, 13]. The release of CGRP from sensory C-fibres can act on adjacent cells, including Langerhans’ cells, macrophages and tissue mast cells [13]. CGRP signalling has a broad inhibitory effect on innate immunity, which is critical in the activation of antigen presenting cells. After binding, CGRP activates cyclic AMP/protein kinase A signalling, which in turn inhibits NF-κB and ICER signalling [13, 17]. As a result, CGRP negatively regulates the production of several cytokines (including IL-12, IFN-γ, TNF⍺, and IL-23), and increases IL-1 and MHC class II expression with effects on dendritic cells, B cells, T cells, and macrophages [13, 17]. Overall, CGRP influences differentiation of CD4 T cells away from the Th1 and Th17 pathways [13, 17]. The various “inflammation dampening” effects of CGRP on components of the immune system are summarised below:

- Langerhans’/tissue dependent dendritic cells: Reduced expression of CD86 and thus antigen presentation, decreased production of IL-1β, TNF⍺ and T_H_1 cytokines. Increased IL-10, IL-4 and T_H_2 cytokines, reduced delayed type IV hypersensitivity response [2, 3].
- Macrophages/Monocytes: Increased IL-10. Decreased CD86 expression, decreased antigen presentation and decreased production of IL-1β, IL-12 and TNF⍺ [2, 3].
- T-Helper (Th17) cells: Increased IL-17, IL-21 and IL-23 expression [2, 3].
- Natural killer cells: Decreased cytolytic activity [2, 3].

Finally, CGRP influences the vascular system in several different ways. It acts on vascular smooth-muscle cells to promote vasodilation, and also exerts an anti-proliferative effect [17]. On endothelial cells, it has a proliferative effect and it may also assist in wound healing through angiogenesis and neovascularisation [17]. Within microvascular endothelial cells, CGRP attenuates leukocyte adhesion [2, 17].

Given these broad effects of CGRP, it is not surprising that we identified migraine patients who developed inflammatory complications after treatment with CGRP mAbs. Of note, we identified patients with complications involving various organ systems (liver, skin, respiratory epithelia) that were de novo, with a clear temporal relationship between exposure and symptom-onset. In each case, there were no other precipitating factors (medication, concurrent infection, etc.) that may have triggered the inflammation. In contrast to the complications seen with other monoclonal antibodies such as checkpoint inhibitors (that result in organ-specific autoimmune disease due to loss of peripheral T-cell tolerance) [18], the clinical presentations in our series are disparate, and are linked only by the addition of CGRP inhibition. We can only speculate as to the driving immunobiology which may link sustained CGRP inhibition and the clinical presentations reported. Importantly, given the paucity of clinical data linking CGRP inhibition with off-target effects, these patients were treated as de novo inflammatory diseases initially, but in several cases maintained remission after tapering of immunosuppressive therapy concurrent with the withdrawal of CGRP mAb, supporting a causative relationship.

Case one of our series is a 56-year-old man with a distant history of rheumatoid arthritis who received a single dose of erenumab for treatment of chronic migraine (CM). Fourteen days after his first dose, he developed biopsy-proven auto-immune hepatitis (AIH). He was treated with prednisolone and azathioprine, and his condition stabilised on treatment and with cessation of erenumab. The temporal relationship between erenumab and the development of AIH is suggestive of a causal association.

Case two is a 67-year-old lady with no history of inflammatory disease who was commenced on erenumab for chronic migraine. She presented after 12 months of treatment with a three-month history of escalating symptoms with blurred vision preceding segmental visual loss in the left eye. Following extensive investigation including MRI, MRA, fluorescein angiography and vitreous biopsy to exclude infection, she was diagnosed with ocular-limited Susac’s syndrome. Erenumab was stopped and her condition stabilised with a combination of intravenous immunoglobulin, prednisolone and mycophenolate. The pathophysiology of Susac’s syndrome, is not completely known, although there are mechanistic studies that identify pathogenic CD8 T-cell-mediated endotheliopathy [19]. Furthermore, histological findings demonstrate endothelial pathology with basement membrane thickening and complement deposition and thrombus formation [20]. In this regard, platelet aggregation has been shown to be inhibited in vitro by CGRP, by increasing platelet cyclic AMP concentrations [21]. Therefore, inhibition of CGRP pathways may promote platelet aggregation and thrombus formation. As discussed previously, an increase in TNF⍺ and reduction of IL-10, would also support a pro-inflammatory immune-phenotype, and the development of Susac’s syndrome.

Case three is a 44-year-old lady with no significant past medical history, apart from chronic migraine. She received one dose of erenumab 70 mg and developed biopsy-proven drug reaction with eosinophilia and systemic symptoms (DRESS) 26 days post treatment. Her condition improved with steroid treatment. The temporal relationship in this case is clear. It should also be noted that DRESS can be an idiosyncratic reaction, such as that seen with carbamazepine. However, an alternative hypothesis implicates the inhibition of CGRP directly in the clinical presentation. It is previously reported that re-activation of herpes viruses, and the subsequent inflammatory response is a significant factor in DRESS [22]. In this context, CGRP has a role in the immune response to viral infection, preventing viral replication and cross-reactivity [10], and in vitro, CGRP gene expression was reduced by intra-nerve *varicella zoster virus* [23]. While speculative, it may be that inhibition of CGRP in this case had a role in re-activation of a herpes virus. In a prospective study of patients with DRESS, a T_H_1 driven response was identified by studying tetramers with viral peptides, with TNF⍺, IFNɣ and IL-2 directed against EBV peptides [24]. As discussed previously, CGRP inhibition would further aggravate this process by increasing TNF⍺ and preventing the down-regulation of IFNɣ.

Case four is a 32-year-old lady with no other significant medical history who received fremanezumab for chronic migraine, after receiving erenumab for six months. After presenting with unilateral hearing loss, she ultimately underwent mastoidectomy, where she was found to have significant paranasal disease. Biopsy demonstrated granulomatous changes and, following investigation she was diagnosed with granulomatosis with polyangiitis (GPA). Fremanezumab was ceased and she was managed with prednisolone and methotrexate.

There are several possible mechanisms by which CGRP inhibition may promote this disease process. In studies of *Moraxella catarrhalis,* CGRP has been shown to decrease leukocyte migration and recruitment in the lung. CGRP inhibition may therefore increase leukocyte recruitment in other disease states such as GPA, contributing to the hyper-activation seen in the disease [25, 26]. Furthermore, induction of T_H_17, releases IL-17 and further liberates TNF⍺ and IL-1β, (all of which are further promoted by CGRP inhibition) leading to hyper-activation of neutrophils, formation of reactive oxygen species, increased lytic enzymes and cytokines that injures vascular endothelial cells and cause the prototypic granuloma formation [26].

Case five is a 20 year-old lady with an existing diagnosis of IgG4 disease (on long-term stable prednisolone), who received galcanezumab for chronic migraine. She developed severe generalised polyarthralgia four days after the first injection, which was temporally related to the medication and improved with cessation of the CGRP mAb. Auto-antibody and inflammatory testing at the time was negative. While less definitely associated with CGRP mAb treatment due to the pre-existing diagnosis of IgG4 disease and long-term immunosuppression, this case is of note as it is the second such case reported in the literature of a polyarthralgia post CGRP inhibition [12]. The temporal onset, subsequent improvement and previous report raises the possibility that the poly-arthralgia may be associated with CGRP.

Case six and seven of our series experienced severe flares of psoriasis or psoriatic arthritis after 1–2 doses of a CGRP mAb. The current understanding of psoriatic pathology is characterised by initiating and maintenance phases. In the initial phase, antimicrobial peptides secreted by keratinocytes stimulate plasmacytoid dendritic cells, promoting myeloid dendritic cell maturation and resultant T_H_1 and T_H_17 differentiation [27]. Activated myeloid dendritic cells migrate to lymph nodes and secrete TNF⍺, IL-12 and IL-23. During the maintenance phase, T_H_17 cytokines promote keratinocyte proliferation, which in turn promote a local inflammatory cascade [27].

Inhibition of CGRP overlaps at several points along this pathological pathway with both pro- and anti-inflammatory effects. CGRP inhibition increases antigen presentation to dendritic cells, and by increasing IL-12 production, promotes T_H_1 differentiation and T_H_17, which drives psoriariform lesions. CGRP inhibition reduces IL-10 production in Langerhans’ cells, and increases IL-1β and T_H_1 cytokine production which could further promote local inflammation. Conversely, CGRP also induces the innate immune response in the skin by increasing IL-8, immune-cell recruitment and antimicrobial protein production [10]. CGRP may also regulate the balance of inflammation and healing in response to cutaneous bacterial infection, with CGRP augmentation promoting *staphylococcus epidermidis* virulence [28], while in a *streptococcus pyogenes* model, inhibition of CGRP improved wound healing [29]. The immune effects of inhibition of CGRP in the skin may therefore be mixed, and a greater understanding is needed of both the role of CGRP and impact of CGRP inhibition in this area [10].

Case eight is a 46-year-old lady who suffered a severe onset of urticarial eczema without any other known cause or triggering factor, 16 months after onset of CGRP mAb treatment for chronic migraine. It is possible that through a similar mechanism to psoriasis, CGRP inhibition may have rendered the patient susceptible to this condition. However, the relatively late onset of the complication may also suggest coincidence or a second inciting factor.

**Fig. 2:**
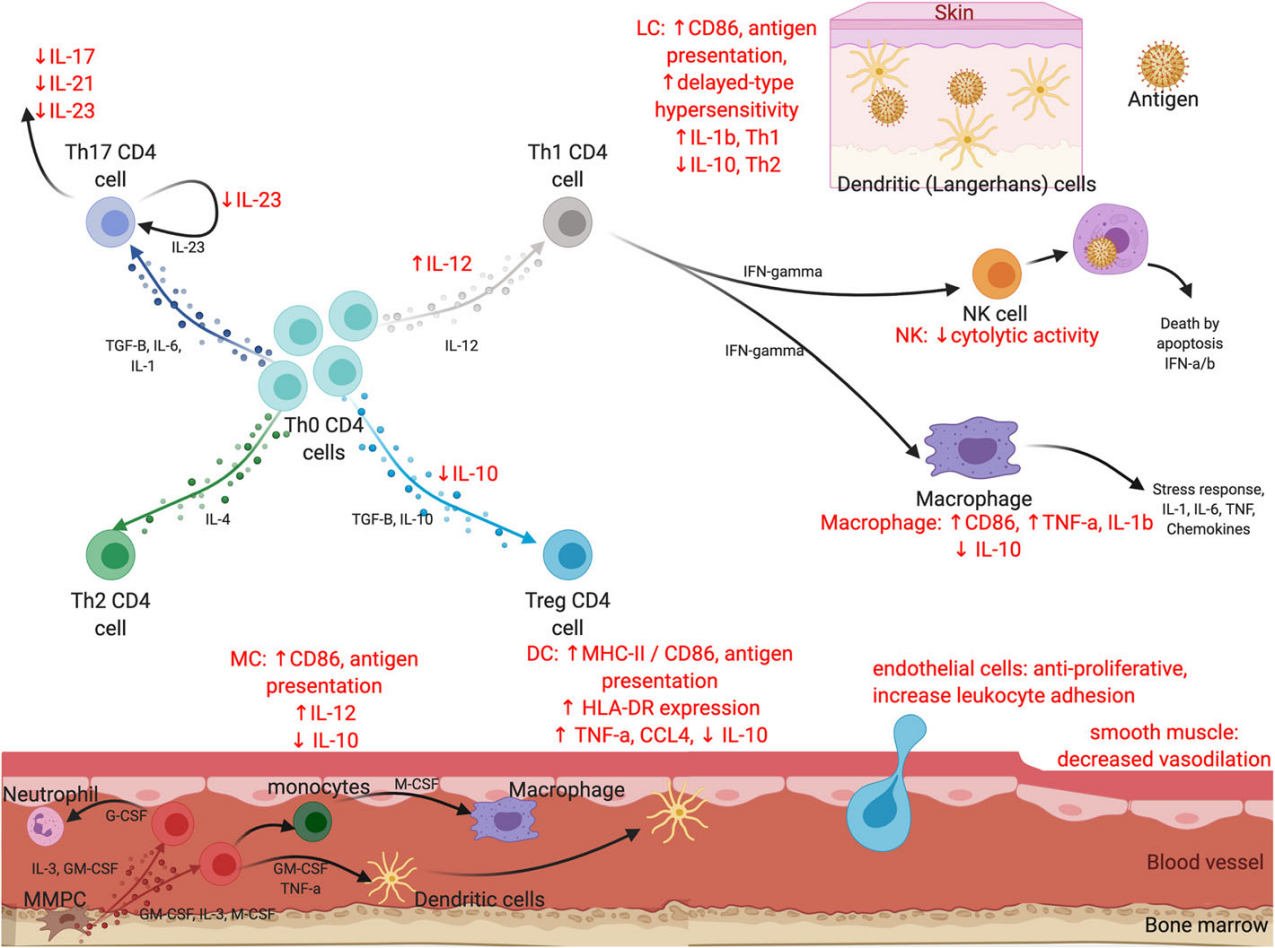
*Potential impact of CGRP inhibition on the immune system.* LC: Langerhans’ cell, NK: Natural killer cell, IFN: interferon, MC: monocyte, DC: dendritic cell, MMPC: myelomonocytic progenitor cell, CSF: colony-stimulating factors. Red text: effect of CGRP inhibition

There are several limitations to this case series. Firstly, biopsy diagnosis was not available for all patients. Secondly, our case series is small considering the number of patient-years of exposure to these medications worldwide. Thirdly, there is no denominator of patients who have been prescribed a CGRP monoclonal antibody in our various clinics, and therefore, the incidence is unestablished. Finally, the possibility of coincidence cannot be discounted, given the well-reported increased frequency of autoimmune disease in younger females. These limitations notwithstanding, given the described temporal and biologically plausible pathological associations, we propose that inhibition of CGRP has a clinically meaningful impact on the immune system in some patients, and the described complications stem from the broad immune effects of CGRP inhibition (Fig. 2).

That these complications have not been reported previously may be due to relatively short trial follow-up and exclusionary criteria. The relative frequency of inflammatory comorbidities in this series raises the question of an ‘at-risk’ cohort not previously recognised. The effect of drug-drug interaction of CGRP inhibiting therapies is not known, and a cautious approach has therefore been proposed [8, 30]. Clinician awareness and the employment of patient registries are critical to determine if there is indeed a sub-group of patients who are at risk of these immunological complications of CGRP inhibition, and whether these patients can be identified through the utilisation of precision medicine, to further improve the safety of these medications [31].

## Data Availability

Not applicable.
